# Impact of the flame retardant 2,2’4,4’-tetrabromodiphenyl ether (PBDE-47) in THP-1 macrophage-like cell function *via* small extracellular vesicles

**DOI:** 10.3389/fimmu.2022.1069207

**Published:** 2023-01-06

**Authors:** Valeria Longo, Noemi Aloi, Elena Lo Presti, Antonino Fiannaca, Alessandra Longo, Giorgia Adamo, Alfonso Urso, Serena Meraviglia, Antonella Bongiovanni, Fabio Cibella, Paolo Colombo

**Affiliations:** ^1^ Institute for Biomedical Research and Innovation, National Research Council of Italy (IRIB-CNR), Palermo, Italy; ^2^ High Performance Computing and Networking Institute, National Research Council of Italy (ICAR-CNR), Palermo, Italy; ^3^ Department of Biomedicine, Neurosciences and Advanced Diagnostics, University of Palermo, Palermo, Italy

**Keywords:** flame retardant, macrophage, extracellular vesiscle, bioinformatic, immunomodulation, microRNA

## Abstract

2,2’4,4’-tetrabromodiphenyl ether (PBDE-47) is one of the most widespread environmental brominated flame-retardant congeners which has also been detected in animal and human tissues. Several studies have reported the effects of PBDEs on different health issues, including neurobehavioral and developmental disorders, reproductive health, and alterations of thyroid function. Much less is known about its immunotoxicity. The aim of our study was to investigate the effects that treatment of THP-1 macrophage-like cells with PBDE-47 could have on the content of small extracellular vesicles’ (sEVs) microRNA (miRNA) cargo and their downstream effects on bystander macrophages. To achieve this, we purified sEVs from PBDE-47 treated M(LPS) THP-1 macrophage-like cells (sEVs^PBDE+LPS^) by means of ultra-centrifugation and characterized their miRNA cargo by microarray analysis detecting the modulation of 18 miRNAs. Furthermore, resting THP-1 derived M(0) macrophage-like cells were cultured with sEVs^PBDE+LPS^, showing that the treatment reshaped the miRNA profiles of 12 intracellular miRNAs. This dataset was studied *in silico*, identifying the biological pathways affected by these target genes. This analysis identified 12 pathways all involved in the maturation and polarization of macrophages. Therefore, to evaluate whether sEVs^PBDE+LPS^ can have some immunomodulatory activity, naïve M(0) THP-1 macrophage-like cells cultured with purified sEVs^PBDE+LPS^ were studied for IL-6, TNF-α and TGF-β mRNAs expression and immune stained with the HLA-DR, CD80, CCR7, CD38 and CD209 antigens and analyzed by flow cytometry. This analysis showed that the PBDE-47 treatment does not induce the expression of specific M1 and M2 cytokine markers of differentiation and may have impaired the ability to make immunological synapses and present antigens, down-regulating the expression of HLA-DR and CD209 antigens. Overall, our study supports the model that perturbation of miRNA cargo by PBDE-47 treatment contributes to the rewiring of cellular regulatory pathways capable of inducing perturbation of differentiation markers on naïve resting M(0) THP-1 macrophage-like cells.

## 1 Introduction

Industrial development has been characterized by the introduction in the environment of several synthetically produced chemical compounds that have led to an ever-greater increase of novel substances potentially capable of affecting human health. Furthermore, with increased environmental pollution, it has been shown that hazardous chemicals can also accumulate, transform, and increase in concentrations in biological systems, exceeding the threshold at which they start to harm health (1, 2). These environmental toxicants can have direct cyto- and/or genotoxicity effects but also induce changes in epigenomic biology that may lead to adverse effects on health (3–6). Recently, several lines of evidence have provided clues about the roles that environmental pollutants can play in the pathophysiology and immunotoxic effects in humans as shown by the increased frequency of some immunological diseases such as immunosuppression, allergies, and autoimmunity (7).

Polybrominated diphenyl ethers (PBDEs) are a large cluster of synthetic compounds (209 congeners) which are listed as persistent organic pollutants (POPs) by the Stockholm Convention due to their well-known resistance to degradation and widespread nature resulting from their hydrophobicity, long-range transportation, and deposition capabilities (ATSDR, 2017 https://www.atsdr.cdc.gov/SPL/). PBDEs are typically used as additives to plastics, therefore they can easily contaminate the environment by leakage. Due to their lipophilicity, they can accumulate in several tissue types as well as matrices such as water, soil, sediment, food, biota, and many animal species (8–10). In this respect, 2,2’4,4’-tetrabromodiphenyl ether (PBDE-47) is one of the most widespread congeners whose presence has been demonstrated in biotic and abiotic environment (11). Several papers have studied the effects of PBDEs on different health issues, including neurobehavioral and developmental disorders, reproductive health, and alterations of thyroid function by working as endocrine disrupting molecules (12, 13). Furthermore, these chemicals can affect cellular homeostasis, and their presence has been linked to ROS production, DNA damage and apoptosis. PBDE-47 also affects immunity in a number of animal species (14, 15), and *in vitro* assays have demonstrated that it can impair innate immune response (16–18).

The immune system is a complex network of components spread all over the body including different organs, immune cells, and immune-active substances (antibodies, lysozymes, complement factors, immunoglobulins, cytokines, etc.), coordinated to accomplish several functions, such as immunologic surveillance, defense, and immunoregulation. The transfer of metabolites and biological information between cells is essential for the coordination of various functions and the regulation of different processes (19), and to achieve this role, immune cells actively communicate through direct interactions or soluble cytokines (20, 21). Recent studies have discovered a new type of cell-to-cell communication in which extracellular vesicles (EVs) work as specific shuttles between donor and recipient cells (22). EVs are a group of diverse membranous component secreted by most cells in extracellular spaces that can be found in almost all biological fluids. These membrane-bound vesicles can transport biomolecules such as proteins, lipids, nucleic acids, small molecules, and receptors. Increasing evidence demonstrates that EVs play important roles in organism development (23), immune response (24), neurons (25), and tissue repair (26). With the discovery of microRNAs (miRNAs) in EVs, and with the demonstration of their transfer to cells, growing interest has been directed to the regulatory roles of EVs. In terms of EV content (cargo), evidence suggests a regulated packaging mechanism for proteins and nucleic acid species (27), although the details of the molecular mechanism behind the packaging remain unclear.

Macrophages are highly plastic cells that can be activated and polarized into classically activated macrophages (M1 macrophages) or alternatively activated macrophages (M2 macrophages) in different microenvironments. Accumulating evidence has shown that M1 and M2 macrophage polarization participates in several innate immune responses but also shapes the adaptive immune response (28). Macrophage-derived EVs have been described, showing that they play an important role in the pathogenesis of inflammatory exacerbation and resolution by interacting with different cell types (16, 29–31). A paradigmatic example of this is the crosstalk between tumoral and immune cells that secrete EVs in the tumor immune microenvironment, modulating immunological activities including macrophage polarization, T cell regulation, and the inhibition of natural killer (NK) cell activity (32, 33).

In this paper, we studied the impact that a widespread environmental toxicant such as PBDE-47 can have on the macrophage’s epigenomic biology. We purified small EVs (sEVs) from PBDE-47 treated M(LPS)- THP-1 macrophage-like cells and characterized their miRNA cargo by microarray analysis. Then, we explored the effect of these PBDE-47-treated THP-1 derived sEVs on naïve M (0) THP-1 macrophage-like cells, looking at intracellular miRNA expression, cytokine production and analysis of some macrophage markers of polarization by flow cytometry.

## 2 Materials and methods

### 2.1 Reagents

PBDE-47 was purchased from Toronto Research Chemicals (cat. T291145) (Ontario, Canada) and dissolved at 25 mM in dimethyl sulfoxide (DMSO) (cat. n. D2650, Sigma-Aldrich, Milan, Italy) as a stock solution. Lipopolysaccharides or LPS (*E. coli* serotype O26:B6) were purchased from Sigma-Aldrich (Milan, Italy).

### 2.2 THP-1 cell line cultures

The human monocytic leukemia THP-1 cell line (ECACC 88081201) was maintained in culture with RPMI 1640 medium (Gibco Life Technologies, Monza, Italy), supplemented with heat inactivated 10% Fetal Bovine Serum (FBS, Gibco Life Technologies, Monza, Italy) and 1% antibiotic (penicillin 5,000 U/mL, Streptomycin sulfate 5,000 µg/mL) (Gibco, Life Technologies, Monza, Italy). THP-1 monocytes were differentiated in M(0) macrophage-like cells (also called “M0 naïve”) by treatment with 200 nM phorbol 12-myristate-13-acetate (PMA, Sigma-Aldrich, Milan, Italy) and incubated for 72 hours at 37°C and 5% CO_2_. The M(LPS) THP-1 phenotype was induced by stimulation with 10 ng/mL of LPS for 24 hours.

### 2.3 M(LPS)-THP-1 derived sEVs separation and analysis

Small EVs were prepared after incubation of the M(0) THP-1 macrophage-like cell line with 3 μM PBDE-47 or 0.0125% DMSO (concentration of the solvent used to get 3 μM PBDE-47 solution considered as control) for 24 hours at 37°C and 5% CO_2_. Then, 10 ng/mL of LPS cells were added to the cells and incubated at 37°C and 5% CO_2_ for a further 24 hours. Supernatants were collected and sEVs were separated according to the gold standard differential ultracentrifugation (dUC) method, as previously described by Longo and co-workers (16). Three independent preparations were used for the assays. The sEVs^DMSO+LPS^ and sEVs^PBDE+LPS^ derived from M (LPS) THP-1 cells were resuspended in RPMI-1640, 1% DMSO and 1% Pen-Strep (Gibco, Life Technologies, Monza, Italy) and stored at -80°C until use. An aliquot of each sample was analyzed by means of Nanoparticle Tracking Analysis (NTA) (NanoSight NS300, Malvern Panalytical, UK) to quantify the sEVs yield produced by M(LPS) THP-1 macrophage-like cells. For each preparation, a mean concentration of approximately 5x10^11^ particles/mL was obtained (see **Supplementary Figure 1** for NTA analysis).

### 2.4 MiRNA purification from sEVs

Purified sEVs^DMSO+LPS^ and sEVs^PBDE+LPS^ were used for miRNA purification. Specifically, miRNAs were extracted from the same number of sEVs^DMSO+LPS^ and sEVs^PBDE+LPS^ (3x10^9^ particles), diluted up to 200 μL with 1X PBS w/o Ca^2+^ and Mg^2+^ and then lysed using 1 mL of QIAzol Lysis Reagent (Qiagen, Milan, Italy). MiRNA purification was performed according to the miRNeasy Serum/Plasma Kit manufacturer’s protocol (Qiagen, Milan, Italy). To control the yield, purity, and integrity of samples, 1µl of a Spike-in mix containing UniSp2 (5’GUACUCGGCUUACGAUCGUAA), UniSp4 (5’GAUGGCAUUCGAUCAGUUCUA) and UniSp5 (5’GAUGCUACGGUCAAUGUCUAAG) miRNAs (Qiagen, Milan, Italy) was added to the samples before the extraction phases. Total RNA, enriched in miRNAs, was eluted in 15µl of H_2_O DNAse/RNAse free, and concentrations were evaluated by Nanodrop analysis (NanoDrop™ One/OneC Microvolume UV-Vis Spectrophotometer, Thermo Fisher Scientific, Monza, Italy).

### 2.5 sEVs^PBDE+LPS^ miRNA profiling

The cDNA synthesis from sEVs^DMSO+LPS^ and sEVs^PBDE+LPS^ miRNAs was performed starting from 250 ng of templates and using the miRCURY LNA^™^ RT kit (Qiagen, Milan, Italy). A mix containing UniSp6 (*5’CUAGUCCGAUCUAAGUCUUCGA*) and *Caenorhabditis elegans* miR-39-3p (*Ce miR39-3p, 5’UCACCGGGUGUAAAUCAGCUUG*) exogenous controls was added to reactions according to the manufacturer’s protocol in a final volume of 20 µl. The retro-transcriptions were performed for 60 minutes at 42°C. Then, the reverse transcriptase enzyme was inactivated for 5 minutes at 95°C. Subsequently, 5 µl of cDNA template was amplified by Real Time analysis (StepOnePlus™ Real Time PCR System, Applied Biosystems) using the miRCURY LNA miRNA Focus PCR Panel (cat. YAHS-201Z, Qiagen, Milan, Italy) and the miRCURY LNA™ SYBR GREEN PCR kit. The Real Time PCR conditions were an initial heat activation step at 95°C for 2 minutes and 40 cycles of two-step PCR, denaturation at 95°C for 10 seconds, annealing/extension at 56°C for 1 minute. The CT data obtained from control (sEVs^DMSO+LPS^) and sEVs^PBDE+LPS^ were analyzed using the Qiagen GeneGlobe miRCURY LNA miRNA PCR Data Analysis software (https://dataanalysis2.qiagen.com/miRCury); data were normalized using the UniSp6 exogenous control (since miRNA housekeeping in THP-1-derived sEVs is not yet known) and the geNorm “Predefined reference miRNA only” function as references. The miRNAs were considered changed between the two groups if the fold change was <0.5 (down-regulated miRNA) or the fold change was >2 (up-regulated miRNA). The miRNAs with a quantification cycle (CT)>35 were considered undetected. The complete list of 84 analyzed miRNAs is reported in **Supplementary Figure 2**.

### 2.6 Culture of M(0) THP-1 macrophage-like cells with sEVs^PBDE+LPS^ and intracellular miRNA profiling

The THP-1 cells were seeded in 6-well plates at the concentration of 5x10^5^ cells/mL and differentiated in M(0) THP-1 naïve macrophage-like cells as described above. Then, the cells were incubated with sEVs^DMSO+LPS^ (control) or sEVs^PBDE+LPS^ in a 1:2 ratio to recipient cells for 24 hours at 37°C and 5% CO_2_. Then, M(0) THP-1 were washed with 1X PBS w/o Ca^2+^ and Mg^2+^ and lysed by adding 350 µl of Qiazol reagent; before the extraction phase, 1µl of UniSp2/4/5 spike-in mix (Qiagen, Milan, Italy) was added to the samples, and total RNA enriched in miRNAs was purified using the miRNeasy Mini Kit (Qiagen, Milan, Italy) following the manufacturer’s protocols. Three independent experiments were run. MiRNAs from treated M(0) THP-1 macrophage-like cells were retro-transcribed after the addition of UniSp6 and Ce-miR39-3p exogenous controls, and the miRNA array analyses were performed using the miRCURY LNA miRNA Focus PCR Panel described above for sEVs miRNA profiling. The CT data obtained from control and treated cells were analyzed using the Qiagen GeneGlobe miRCURY LNA miRNA PCR Data Analysis software tool, and the expression values were reported as fold change. Normalization was performed by means of the geNorm (only pre-defined reference miRNA) tool calculating a normalization factor based on several reference miRNAs (hsa-miR-151a-5p, has-SNORD4, has-SNORD38B, has-SNORD49A and U6 snRNA) provided in the microarray panel.

### 2.7 Bioinformatic analysis of miRNA-target interactions

To obtain interaction analyses, a bioinformatic approach was carried out to identify the biological pathways that could be involved in this study. Our analysis comprised two steps: identification of the validated genes targeted by deregulated miRNAs and extraction of the biological pathways affected by these target genes. Regarding the first step, a set of experimentally validated miRNA-target interactions was obtained from up- and down-regulated miRNAs, using the relationships reported in the miRTarBase (release 9.0) database (34). The miRTarBase repository collects more than 2,200k experimentally validated miRNA target interactions (MTIs) in 37 species, extracted from 13k manually curated articles. Starting from MTIs involving deregulated miRNAs, the target genes interacting with a significant number of miRNAs were considered for the next step since they had a greater chance of being dysregulated. For this purpose, a graph having miRNAs and targets as nodes and their interactions as edges was built. Only node targets with a higher degree than a fixed threshold were considered for the rest of this work. Regarding the second step, identification of statistically relevant biological pathways was performed from a previously extracted list of putative dysregulated target genes. For this objective, the Reactome Pathway Analysis (ReactomePA) package (v1.40.0) (35) or R programming language was used since it allowed us to perform the enrichment analysis by considering the pathways collected in the Reactome platform (36). At this point, a list of biological pathways involved with the previously selected target genes was obtained and then clustered according to their biological relationships, as defined in the Reactome pathway hierarchy document. For each cluster, a representative pathway maintaining all the target genes of the cluster was selected.

### 2.8 Total RNA preparation and real time analysis from naïve M(0) THP-1 macrophage-like cells treated with sEVs^PBDE+LPS^

Total RNAs from naïve M(0) THP-1 macrophage-like cells treated with sEVs^PBDE+LPS^ were extracted according to the RNeasy Mini Kit (Qiagen, Milan, Italy) manufacturer’s protocol (n=4). The cDNAs were retro-transcribed using the QuantiTect Reverse Transcription Kit (Qiagen, Milan, Italy) using 2.5 µg/reaction of RNA template. The cDNA was diluted up to 100 μl and real time analyses were performed using a Step OnePlus™ Real Time PCR System (Applied Biosystems, Monza, Italy) and SYBR Green technology as previously described (16). The primer sequences (Qiagen, Milan, Italy) used for human cytokines were: IL-6 (Interleukin 6, NM_000600), TNF-α (Tumor necrosis factor alpha, NM_000594) and TGF-β (Transforming Growth Factor-β, NM_000660); the housekeeping gene used in all analyses was ACT (human actin beta, NM_001101).

### 2.9 Flow cytometry analysis of the -M(0) THP-1 macrophage-like cells treated with sEVs

To study the functional performance of sEVs^PBDE+LPS^, we incubated resting M(0) THP-1 macrophage-like cells with equimolar concentrations of sEVs^DMSO+LPS^ and sEVs^PBDE+LPS^ for 24 hours. After this time, cells were gently detached using Accutase solution (Sigma-Aldrich, Milan, Italy) and incubated at 37°C and 5% CO_2_ for almost 5’. Each gating strategy analyzed the selected macrophage population for CD14/CD11b expression. Resting M(0) THP-1 macrophage-like cells treated with different vesicles were stained for live/dead discrimination using Invitrogen Live/Dead (Invitrogen, Carlsbad, CA, USA). Fc receptor blocking was performed with human immunoglobulin (Sigma-Aldrich, Milan, Italy) (3 mg/ml final concentration), followed by surface staining with different fluorochrome-conjugated antibodies to study the expression of some markers below described. The fluorescein isothiocyanate (FITC), phycoerythrin (PE)-, PE-Cy5-, allophycocyanin (APC)-, phycoerythrin-Cy7 (PECy7)-, allophycocyanin- Cy7 (APC-Cy7)-conjugated monoclonal antibodies (mAbs) used to characterize the entire population were the following: anti-CD14, anti-CD11b, anti-CD209, anti-CD38, anti-CD80, anti-HLA-DR, anti-CCR7, anti-CD45. The expression of surface markers was determined by flow cytometry on a FACSCanto II (BD Biosciences, Milan, Italy) using FlowJo software (BD Biosciences, Milan, Italy). The gating strategy involved progressively measuring total viable cells only. For each sample, 20,000 nucleated cells were acquired, and values were expressed as the percentage of viable differentiated THP-1 as gated by forward and side scatter.

### 2.10 Statistical analysis

Flow cytometry analysis was performed by using the Mann-Whitney test and two-tailed P value. All values were expressed as mean ± SD for each group. Differences were considered statistically significant when p-values were ≤ 0.05 (*). During the pathway enrichment analysis, the selection of biologically relevant pathways was statistically assessed by calculating the p-value using the hypergeometric distribution (34). To avoid type I errors (false positives) and provide a more accurate statistical analysis, a correction test using the Benjamini-Hochberg (BH) method (37) (beyond a cut off of 0.05) was applied to obtain an adjusted p-value.

## 3 Results

### 3.1 PDBE-47 modulates sEVs^PBDE+LPS^ miRNA cargo in M(LPS) THP-1 macrophage-like cells

**Figure 1:**
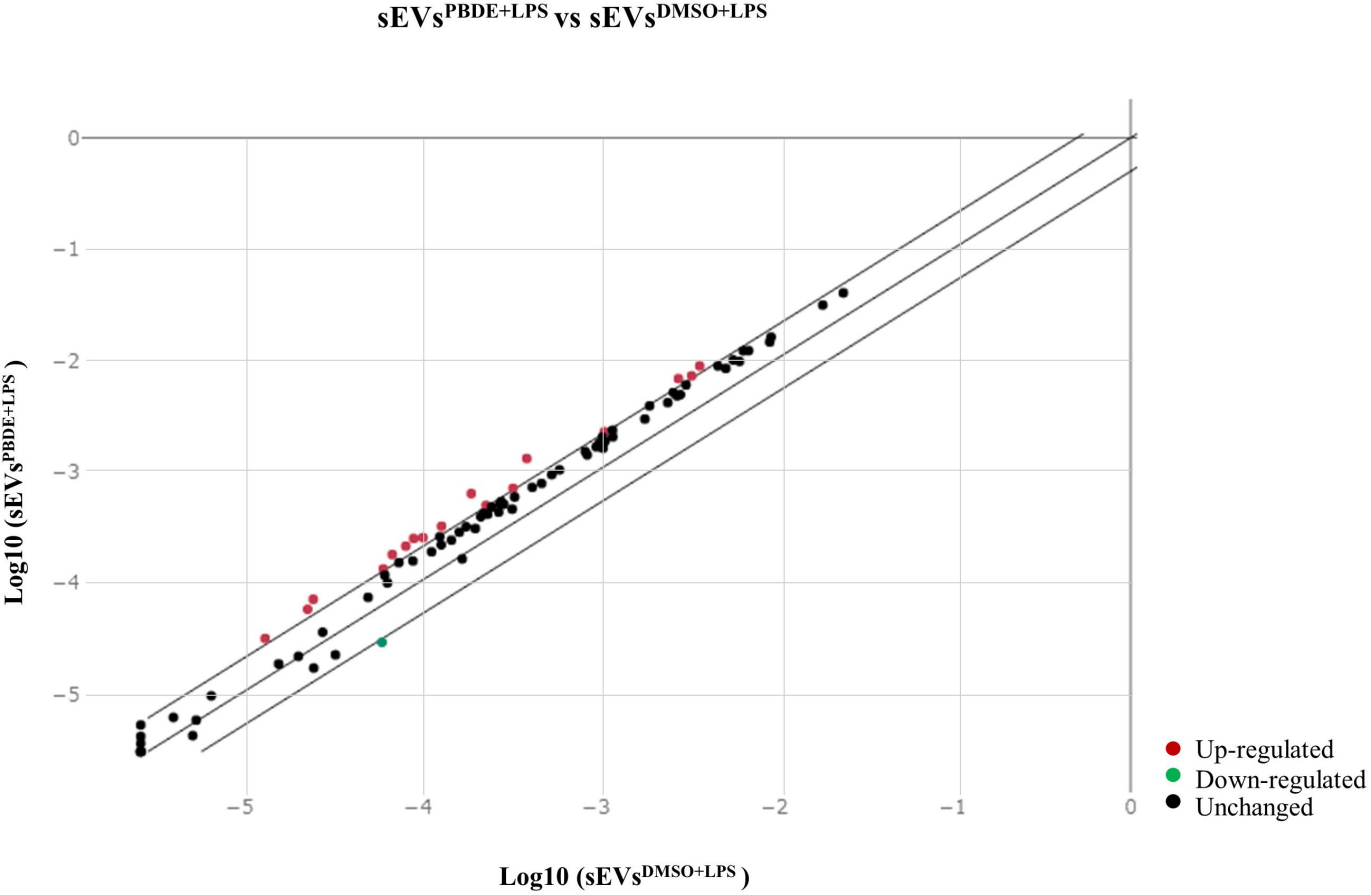
Scatterplot of miRNA microarray analysis. CT data from samples (sEVs^PBDE+LPS^) and control (sEVs^DMSO+LPS^) were analyzed with the GeneGlobe miRCURY LNA miRNA PCR Data Analysis tool (https://dataanalysis2.qiagen.com/miRCury). MiRNAs were considered down-regulated if the fold change was <0.5 (green dot) and up-regulated if the fold change was >2 (red dots); in black are reported the unchanged miRNAs with respect to control (sEVs^DMSO+LPS^). Normalization for the fold change calculation was performed using UniSp6 exogenous control and the geNorm (Predefined reference miRNA only) function.

The conditioned culture media of M(LPS) THP-1 macrophage-like cells, stimulated with PBDE-47 or DMSO, were used to purify EVs according to a gold-standard method (38, 39). In this manuscript, we focused on sEVs sub-populations (sEVs^DMSO+LPS^ and sEVs^PBDE+LPS^, respectively) purified from 3 experimental replicates. The array data used for the profiling of the cargo miRNAs (Supplementary Figure 1) showed the up-regulation of 17 independent miRNAs (**Tables 1**, **2**): hsa-miR-142-5p (fold change=2.38), hsa-miR-27b-3p (fold change=2.05), miR-103a-3p (fold change=3.22), miR-185-5p (fold change=2.51), miR-181b-5p (fold change=2.09), miR-30e-5p (fold change=2.34), miR-200c-3p (fold change=2.41), miR-15a-5p (fold change=2.06), miR-29a-3p (fold change=2.07), hsa-miR-19a-3p (fold change=2.11), miR-143-3p (fold change=2.50), miR-19b-3p (fold change=2.37), miR-17-5p (fold change=2.39), miR-22-3p (fold change=2.65), miR-29c-3p (fold change=2.20), miR-7-5p (fold change=2.28) and miR-100-5p (fold change=2.76); it also showed the down-regulation of miR-122-5p expression (fold change=0.47). Altogether, these results highlighted that PBDE-47 treatment was able to increase the uploading of a large set of miRNAs into M(LPS) THP-1 macrophage-like cells derived sEVs. A graphical representation of sEVs miRNA expression is reported in **Figure 1**.

**Table 1:** Summary of sEV^PBDE+LPS^ array data compared to control (sEV^DMSO+LPS^).

Total number of analyzed miRNAs	84
**Up-regulated miRNAs**	**17**
**Down-regulated miRNAs**	**1**
**Undetected miRNAs**	**10**
**miRNAs equal to Control (sEV^DMSO+LPS^ )**	**56**

Cut-off values:

• Up-regulation Fold Change >2;

• Down-regulation Fold Change <0.5.

**Table 2:** Fold change values of dysregulated miRNAs in sEV^PBDE+LPS^.

miRNA ID	Fold Change
hsa-miR-142-5p	2.38
hsa-miR-27b-3p	2.05
hsa-miR-103a-3p	3.22
hsa-miR-185-5p	2.51
hsa-miR-181b-5p	2.09
hsa-miR-30e-5p	2.34
hsa-miR-200c-3p	2.41
hsa-miR-15a-5p	2.06
hsa-miR-29a-3p	2.07
hsa-miR-19a-3p	2.11
hsa-miR-143-3p	2.50
hsa-miR-19b-3p	2.37
hsa-miR-17-5p	2.39
hsa-miR-22-3p	2.65
hsa-miR-29c-3p	3.19
hsa-miR-122-5p	0.47
hsa-miR-7-5p	2.28
hsa-miR-100-5p	2.76

### 3.2 sEVs^PBDE+LPS^ modulate the intracellular miRNA profile in naïve M(0) THP-1 derived macrophage-like cells

**Figure 2:**
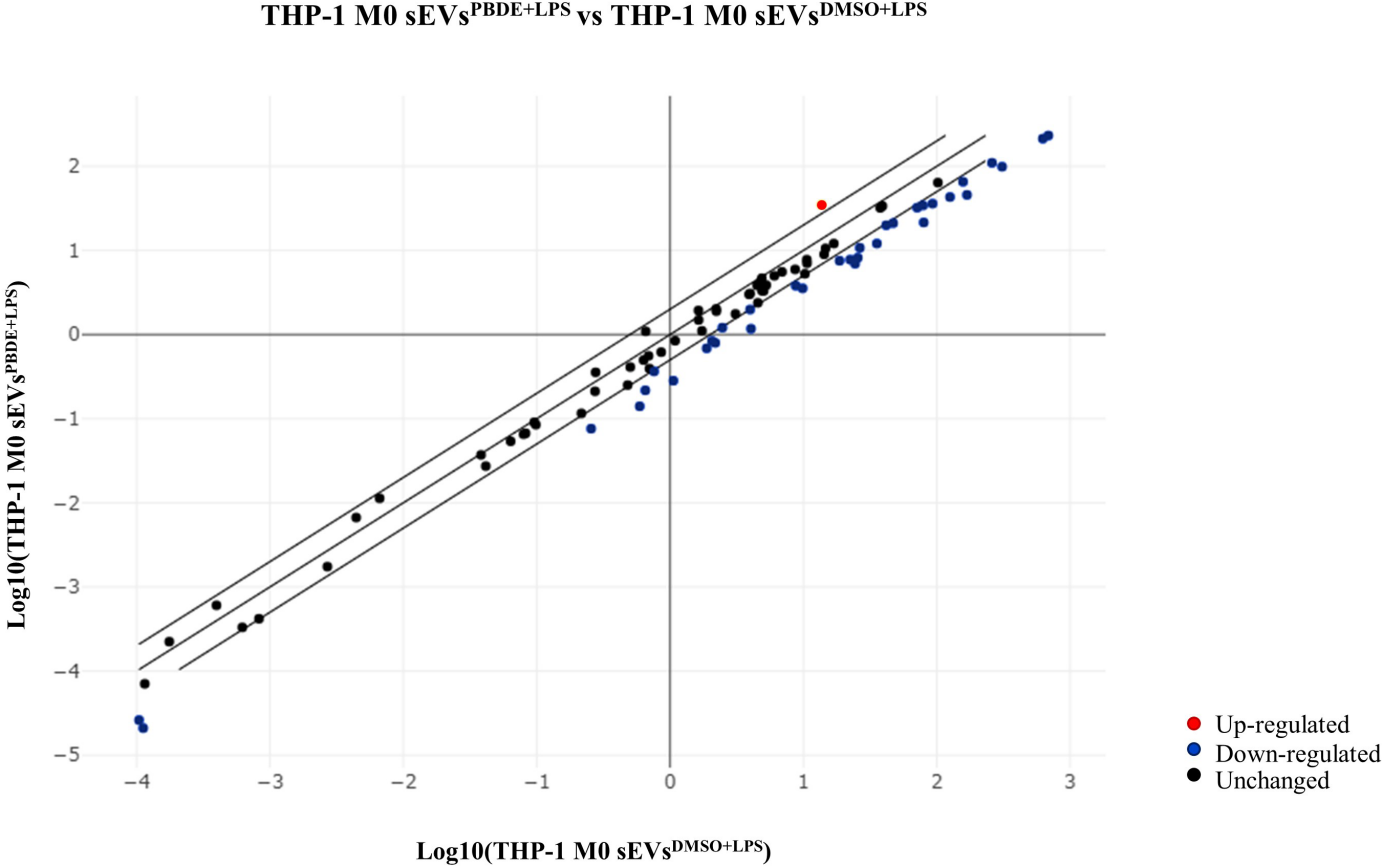
Scatterplot of miRNA expression in M(0) THP-1 macrophage-like cells treated with sEVs^PBDE+LPS^. CT values obtained from three independent experiments (M(0) THP-1 macrophage-like treated with sEVs^PBDE+LPS^ vs controls) were analyzed using the GeneGlobe miRCURY LNA miRNA PCR Data Analysis tool. Blue dots show down-regulated miRNAs (fold change <0.5), red is for up-regulated miRNA (fold change >2), and black indicates unchanged miRNAs with respect to control cells.

In order to simulate the distal cell-to-cell communication, we investigated the effects of sEVs^PBDE+LPS^ on resting M(0) THP-1 macrophage-like cells. For this purpose, sEVs^PBDE+LPS^ or sEVs^DMSO+LPS^ (control) were incubated with naïve M(0) THP-1 macrophage-like cells for 24h. Intracellular miRNAs were isolated from three independent experiments, and microarray analyses were performed using the miRNA Panel described above for sEVs miRNA profiling (**Supplementary Figure 1**). The array data analyses were carried out using the Gene Globe Data Analysis Center. A total of 12 intracellular miRNA were dysregulated by sEVs^PBDE+LPS^ treatment (**Table 3**). The scatterplot reported in **Figure 2** highlights: 11 down-regulated miRNAs (blue dots), hsa-miR-194-5p (fold change= 0.297), hsa-miR-374a-5p (fold change= 0.284), hsa-miR-23a-3p (fold change= 0.423), hsa-miR-126-3p (fold change=0.289), hsa-miR-195-5p (fold change= 0.236), hsa-miR-30d-5p (fold change= 0.403), hsa-miR-222-3p (fold change= 0.434), hsa-miR-186-5p (fold change= 0.406), hsa-miR-196b-5p (fold change= 0.362), hsa-miR-140-3p (fold change= 0.358), hsa-miR-100-5p (fold change= 0.480), as well as the up-regulated hsa-miR-142-3p (red dot, fold change= 2.55).

**Table 3:** Results of dysregulated miRNA in M0 THP-1 treated with sEV^PBDE+LPS^.

miRNA ID	Fold Change
hsa-miR-142-3p	2.55
hsa-miR-194-5p	0.297
hsa-miR-374a-5p	0.284
hsa-miR-23a-3p	0.423
hsa-miR-126-3p	0.289
hsa-miR-195-5p	0.236
hsa-miR-30d-5p	0.403
hsa-miR-222-3p	0.434
hsa-miR-186-5p	0.406
hsa-miR-196b-5p	0.362
hsa-miR-140-3p	0.358
hsa-miR-100-5p	0.480

### 3.3 *In silico* analysis to identify putative biological processes and pathways

**Figure 3:**
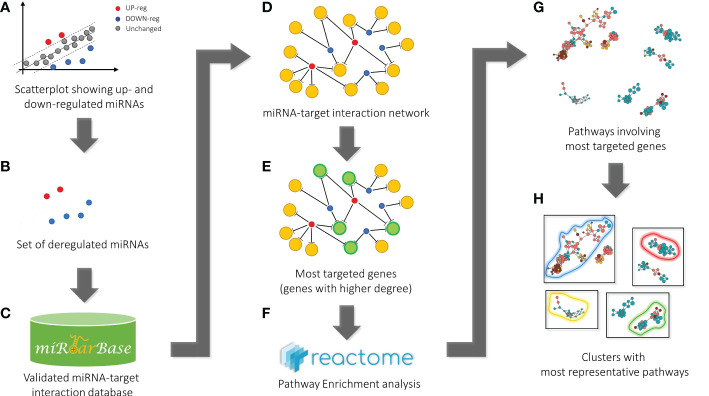
Bioinformatic workflow for pathway enrichment analysis workflow. Starting from the scatterplot showing the analyzed miRNA expressions **(A)**, the set of dysregulated miRNAs (blue and red points) was considered **(B)** to get all the validated miRNA-target pairs from the miRTarBase database **(C)**. At this point, the network containing all the miRNA-target interactions was defined **(D)**. Then, the most targeted genes (green circles), i.e., genes targeted by at least a significant number of miRNAs (genes with a higher degree in the network) **(E)**, were selected for the pathway enrichment analysis according to the Reactome repository **(F)**. Finally, the most statistically significant pathways were identified **(G)** and clustered according to hierarchical relationships defined in the Reactome database. The representative pathways of each cluster (here bounded by colored curves) are given as a result **(H)**.

**Figure 4:**
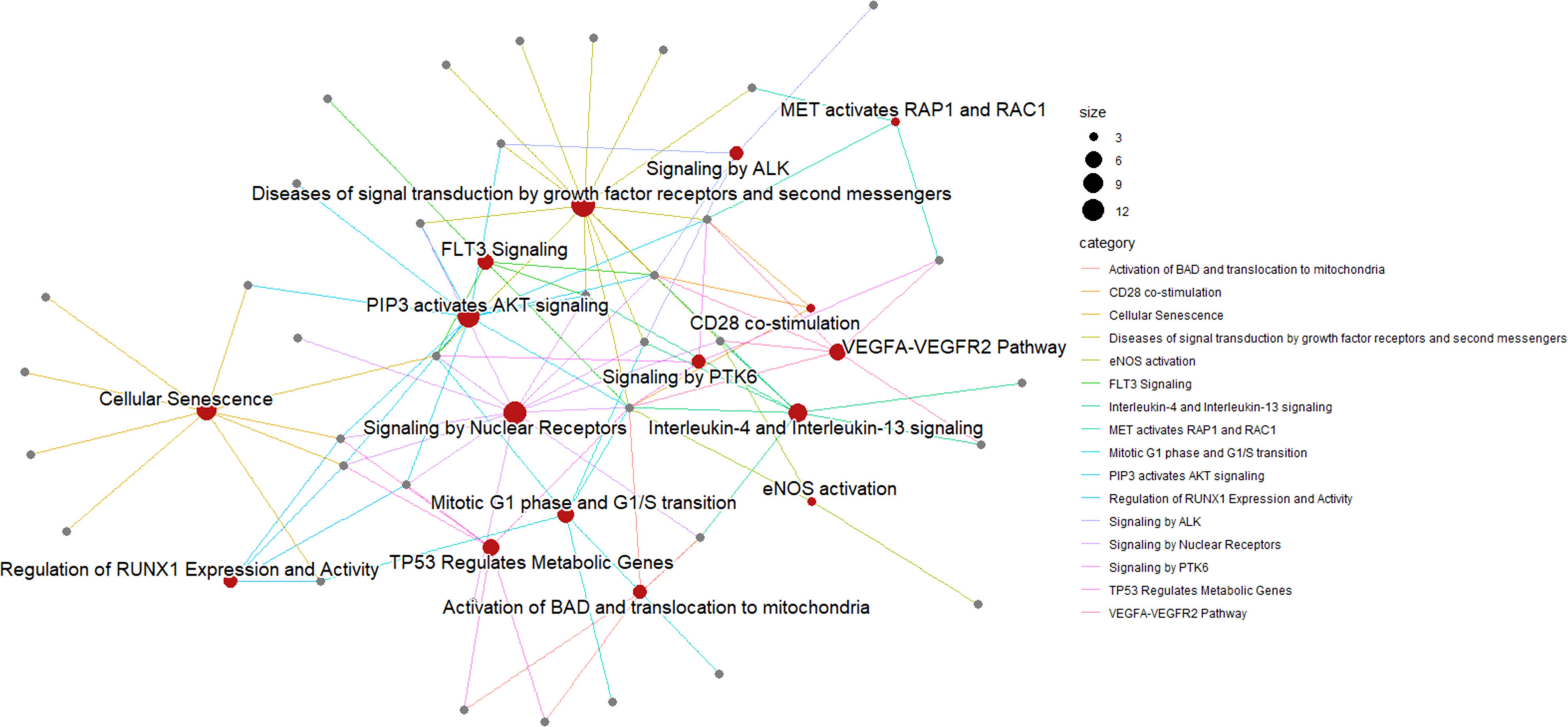
Pathway enrichment analysis of M(0) THP-1 macrophage-like treated with sEV^PBDE+LPS^. A network represents the result of the pathway enrichment analysis. Here, red nodes are pathways, and grey nodes are genes. Links indicate interaction among pathways and genes; each gene can interact with more than one pathway. As shown on the right of the figure, the size of each red node is related to the number of genes contained in the pathway, whereas the color of each link depends on the specific biological pathway.

We used the list of the 12 intracellular M(0) THP-1 macrophage-like cells modulated miRNAs identified in the microarray assay (see **Table 3**) to find biological pathways that could be influenced by co-expressed miRNAs, exploiting details about their experimentally validated putative gene targets. With this aim, we used the miRTarBase repository (http://mirtarbase.mbc.nctu.edu.tw/) (40), which contains a manually curated miRNA-target interaction database, where we identified a gene target set associated with our 12 dysregulated miRNAs. **Figure 3** shows the bioinformatic workflow we designed for this purpose. This set contains 3036 putative targets, most of which (i.e., 2390 genes) interact with a unique miRNA. To add consistency to the *in silico* analysis, we considered those targets with a degree of ≥ 3 miRNAs in the following steps since it statistically gives higher reliability of putative targeted genes. Indeed, we know *in silico* analysis cannot ensure that validated MTIs occur in this specific study. We selected 134 genes for the pathway enrichment analysis according to this criterion. As reported in **Table 4**, the set of genes was composed of 7 genes targeted by 5 miRNAs, 21 genes targeted by 4 miRNAs, and 106 genes targeted by 3 miRNAs. At this point, we performed the pathway enrichment analysis using the “ReactomePA” library (36), obtaining 87 statistically relevant pathways with an adjusted p-value ≤ 0.05. Then, we clustered pathways according to their hierarchical relationships as defined by the Reactome database, obtaining a list of 16 representative pathways of interest. **Table 5** contains the list of representative pathways, sorted by p-value. **Figure 4** provides us with a quick overview of the major biological pathways identified and how they relate to one another. The relationships among enriched pathways clearly show how they are strictly connected in terms of shared target genes; in particular, AKT1 is the most shared gene since it is contained in 12 pathways, i.e., 75% of the representative pathways (see the last column of **Table 5** for the complete list of involved target genes). The most statistically relevant pathway is “Signaling by Nuclear Receptors”, with a p-value of 1.438E-06 and 13 involved genes, whereas the second is “PIP3 activates AKT signaling”, with a p-value of 2.684E-06 and 12 involved genes. Afterwards, we found: “Interleukin-4 and Interleukin-13 signaling” pathway, with a p-value equal to 3.708E-06 and 8 involved genes; “Activation of BAD and translocation to mitochondria” pathway, with a p-value of 6.384E-06 and 4 involved genes; “Regulation of RUNX1 Expression and Activity” pathway, with a p-value equal to 1.09863E-05 and 4 involved genes; “FLT3 Signaling” pathway, with a p-value equal to 1.65405E-05 and 5 involved genes; “Diseases of signal transduction by growth factor receptors and second messengers” pathway, with a p-value of 1.71364E-05 and 14 involved genes; “Cellular Senescence” pathway, with a p-value equal to 4.55404E-05 and 9 involved genes; “Signaling by ALK” pathway, with a p-value equal to 7.58741E-05 and 4 involved genes; “MET activates RAP1 and RAC1” pathway, with a p-value of 9.52706E-05 and 3 involved genes; “TP53 Regulates Metabolic Genes” pathway, with a p-value equal to 9.65964E-05 and 6 involved genes; “eNOS activation” pathway, with a p-value equal to 1.631E-04 and 3 involved genes; “VEGFA-VEGFR2 Pathway” pathway, with a p-value of 1.974E-04 and 6 involved genes; “Signaling by PTK6” pathway, with a p-value of 1.146E-03 and 4 involved genes; “Mitotic G1 phase and G1/S transition” pathway, with a p-value equal to 1.719E-03 and 6 involved genes; “CD28 co-stimulation” pathway, with a p-value equal to 2.749E-03 and 3 involved genes.

**Table 4:** Degree of putative target genes in the miRNA-target interaction network.

N. of targeted genes	Degree of genes (N. of miRNAs targeting the same gene)
7	5
21	4
106	3
512	2
2390	1

**Table 5:** Representative pathways identified by the Reactome-PA database.

Reactome Pathway ID	Pathway Description	Gene Ratio (miRNA target / pathway genes)	p-value	Target Gene List
R-HSA-9006931	Signaling by Nuclear Receptors	13/299	1.44E-06	AKT1/BCL2/CDKN1B/ESR1/FASN/FOXO3/HSP90A A1/MYC/PIK3R1/AGO1/AGO2/TNRC6A/MYLIP
R-HSA-1257604	PIP3 activates AKT signaling	12/267	2.68E-06	AKTI/XIAP/CDKNIB/ESR1/FOXO3/PIK3R1/RACI/ FRS2/AGO1/AGO2/TNRC6A/CBX2
R-HSA-6785807	Interleukin-4 and Interleukin-13 signaling	8/108	3.71E-06	AKTI/BCL2/FOXO3/HSP90AA1/MYC/PIK3R1/VCA MI/VEGFA
R-HSA-111447	Activation of BAD and translocation to mitochondria	4/15	6.38E-06	AKTI/BCL2/YWHAE/YWHAH
R-HSA-8934593	Regulation of RUNX1 expression and activity	4/17	1.10E-05	CDK6/AGO1/AGO2/TNRC6A
R-HSA-9607240	FLT3 signaling	5/38	1.65E-05	AKTI/CDKNIB/FOXO3/PIK3R1/SOCS6
R-HSA-5663202	Diseases of signal transduction by growth factor receptors and second messengers	14/433	1.71E-05	AKTI/CDKNIB/ESRI/FOXO3/HSP90AA1/KIF5B/FZ D6/MYC/PIK3R1/RACI/RAPIB/MIB1/SPRED1
R-HSA-2559583	Cellular senescence	9/197	4.55E-05	ATM/CDK6/CDKNIB/HMGA1/HMGA2/AGO1/MIN K1/TNRC6A/CBX2
R-HSA-201556	Signaling by ALK	4/27	7.59E-05	PRDM1/MYC/PIK3R1/FRS2
R-HSA-8875555	MET activates RAP1 and RACI	3/11	9.53E-05	CRK/RACI/RAPIB
R-HSA-5628897	TP53 regulates metabolic genes	6/87	9.66E-05	AKTI/YWHAE/YWHAH/AGO1/AGO2/TNRC6A
R-HSA-203615	eNOS activation	3/13	0.00016	AKT1/HSP90AA1/DDAH1
R-HSA-4420097	VEGFA-VEGFR2 pathway	6/99	0.0002	AKT1/CRK/HSP90AA1/PIK3R1/RACI/VEGFA
R-HSA-8848021	Signaling by PTK6	4/54	0.00115	AKT1/CDKNIB/CRK/RACI
R-HSA-453279	Mitotic G1 phase and G1/S transition	6/149	0.00172	AKTI/CDK6/CDKNIB/MYC/RRM2/WEE1
R-HSA-389356	CD28 co-stimulation	3/33	0.00275	AKTI/PIK3RI/RACI

### 3.4 Effect of sEVs^PBDE+LPS^ on naïve M(0) THP-1 derived macrophages cytokine expression

To study the effect of the sEVs^PBDE+LPS^ treatment on M1 and M2 polarization, we decided to analyse the expression level of the cytokines IL-6, TNF-α and TGF-β on naïve M(0) THP-1 macrophage-like cells by real time PCR analysis. The quantitative analysis showed no differences in the cytokine transcription between control and sEVs^PBDE+LPS^ after 24 hours of incubation. (**Supplementary Figure 3**).

### 3.5 Immuno-characterization of PMA differentiated M(0) THP-1 macrophage-like cells treated with sEVs^PBDE+LPS^

**Figure 5:**
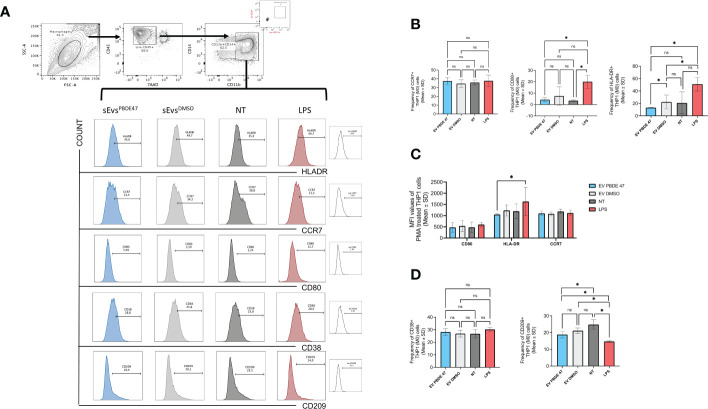
Immuno-characterization of M(0) THP-1 macrophage-like treated with sEVs^PBDE+LPS^ Macrophage sEVs^PBDE+LPS^ on resting M(0) THP-1 macrophage-like affecting expression of surface markers of macrophage polarization by flow cytometry analysis. **(A)** gating strategy analyzing the selected macrophage population for CD14/CD11b expression in each experimental condition. **(B)** Cumulative analysis of resting M(0) THP-1 macrophage-like treated with macrophage sEVs^PBDE+LPS^, or sEVs^PBDE+LPS^, indicated percentage of the CD14/CD11b cells expressing CCR7, CD80 and HLA-DR. Untreated cells (NT) and LPS were considered as experimental control condition. **(C)** Cumulative analysis of the Mean Fluorescence Intensity (MFI) of resting M(0) THP-1 macrophage-like treated with macrophage sEVs^PBDE+LPS^ expressing CCR7, CD80 and HLA-DR. **(D)** Cumulative analysis of resting M(0) THP-1 macrophage-like treated with macrophage sEVs^PBDE+LPS^, or only DMSO, indicated percentage of the CD14/CD11b cells expressing CD38 and CD209. Untreated cells (NT) and LPS were considered as experimental control condition. Non statistical significance is indicated as ns. *P ≤ 0.05 by nonparametric Mann-Whitney test, unpaired and one-tailed with confidential interval 95%.

To evaluate whether sEVs^PBDE+LPS^ have some immunomodulatory activity, we stained naïve M(0) THP-1 macrophage-like cells after incubation with sEVs^DMSO+LPS^ and sEVs^PBDE+LPS^, as described in the Material and Method section, and then we analyzed them by flow cytometry. Considering that the expression of many macrophage markers is known to change in response to stimuli, we chose markers that can reflect commonly studied macrophage phenotypes. For instance, the markers CD11b and CD14 decrease in expression in M(0) macrophages, while CD209 and CD80 increase in expression relative to resting M(0) macrophage. Conversely, stimulated LPS-macrophages decreased CD209 but increased CD38 and CD80, as demonstrated in several studies (41). Based on these concepts and considering that recently CD38 and CD209 were evaluated as differential markers related to the M1 and M2-like phenotypes (42), we evaluated whether a low dose of PBDE-47 can affect immunological synapses through vesicles released by M(LPS) THP-1 macrophage-like cells) (sEVs^PBDE+LPS^). As shown in **Figure 5A**, double positive CD14/CD11b THP-1 cells were evaluated for expressing different markers: CD38, CD209, CCR7, CD80, HLA-DR upon *in vitro* treatment with sEVs^PBDE+LPS^, and using DMSO, LPS and Untreated cells as controls.

**Figure 5B** demonstrates that PMA differentiated M(0) THP-1 macrophage-like cells treated with sEVs^PBDE+LPS^ decreased HLA-DR expression when compared with LPS-stimulated cells and sEVs^DMSO+LPS^ (Means ± SD were 13.13±0.65, 51.07±11, 22.37±11.06, respectively), leading to the hypothesis that the decreasing level of HLA-DR was exclusively determined by PBDE-47. The latter result was significantly confirmed by MFI (Mean Fluorescence Intensity) analysis. Differently, cells positive for CD80 were expressed at very low levels compared to LPS control, even though the same decreased level was common in DMSO. We observed a low increase in CCR7 levels in M(0) THP-1 macrophage-like cells treated with sEVs^PBDE+LPS^ than in LPS, but with nonstatistical significance.

Among the several activation macrophage-subtypes, the best characterized are the “classically activated” (also termed M1) and “alternatively activated” (also termed M2) macrophages. Recently, it has been published that among all markers usually expressed on macrophages-subsets, CD38 and CD209 could be used to define the M1 and M2 phenotypes (42). **Figure 5C** shows the cumulative data of the relative expression of these markers by MFI analysis.

CD38 is considered a marker of cell activation and, for instance, Gram-negative bacterial cell wall LPS induces CD38 expression on macrophages. When (M0) THP-1 macrophage-like cells were treated with sEVs^PBDE+LPS^, there was a weak decrease in expression compared to LPS and non-significant variation compared to untreated and DMSO treated cells. In addition, the MFI analysis demonstrated no changes in expression (data not shown). On the contrary, CD209^+^ antigen (considered as characteristic of the M2-like phenotype) was significantly more expressed on sEVs^PBDE+LPS^-treated (M0) THP-1 macrophage-like cells than upon LPS, but less with respect to untreated cells (Means ± SD were 13.4 ±1.57, 10.50±0.52 18.63±2.2, respectively), indicating a possible intervention of PBDE-47 in macrophage polarization (**Figure 5D**).

## 4 Discussion

In the last few decades, increasing attention has been paid to environmental pollution as one of the most serious problems affecting human health. Environmental epidemiology has aimed to characterize how single, or mixtures of exposures are associated with one or several health outcomes, but it is not sufficient to characterize the associated biological effects (43). Environmental pollutants has drawn much attention since they can come into contact with immune cells *via* the bloodstream, raising concerns about its immunomodulating effects. Our previously published data demonstrated that PBDE-47 treatment can affects the expression of cytokines (IL-1β, TNF-α and IL-6) that are recognized to be essential for innate inflammatory response and microbicidal capacity (16, 17). Moreover, we were able to show that PBDE-47 can modulate the expression of some specific intracellular microRNAs involved in the inflammasome regulation and interfere with the biogenesis of sEVs (16). Indeed, all the immune cell types that participate in the inflammatory reaction can secrete sEVs, which in turn have multiple roles in inflammatory processes. Circulating sEVs may form a signaling network where various cells of the immune system can exchange information, coordinating homeostasis and alerting the immune system upon sensing danger. sEVs can remain associated with the pericellular matrix (i.e., promoting the clearance of cell debris and tissue repair), but they can also travel to distant recipient cells *via* blood-mediated transport (44). Perturbation on such transfer of information to neighboring or distant cells can have effects on the amplification of inflammatory signaling and/or induced pathological responses (45). These observations have left open a further issue addressed by this study regarding to the role that sEVs purified from PBDE-47 treated M(LPS) THP-1 macrophage-like cells can have on the activity and polarization of resting M(0) THP-1 cells with a special focus on miRNA expression.

To try to understand some of mechanisms behind the effects induced by the PBDE-47 on innate immune response, we decided to study the impact of the flame retardant on the miRNA cargo in sEVs derived from M(LPS) THP-1 macrophage-like cell line analyzing the expression levels of 84 selected miRNAs loaded in sEVs^PBDE+LPS^. We observed that PBDE-47 treatment can induce the uploading of 17 cargo miRNAs (miR-142-5p, miR-27b-3p, miR-103a-3p, miR-185-5p, miR-181b-5p, miR-30e-5p, miR-200c-3p, miR-15a-5p, miR-29a-3p, miR-19a-3p, miR-143-3p, miR-19b-3p, miR-17-5p, miR-22-3p, miR-29c-3p, miR-7-5p, and miR-100-5p) and the downloading of miR-122-5p. To the best of our knowledge, this is the first study profiling sEVs miRNA content upon treatment with a flame retardant in immune cells. Changes in miRNA expression levels in sEVs under different physiological and/or pathological conditions have already been described (46–48). For instance, in sepsis, EVs have been shown to have both pro- inflammatory and anti- inflammatory roles (49), depending on the donor cell type and microenvironmental stimuli inducing the uploading of different cargo molecules (50, 51) suggesting that EVs wield “a double-edged sword” in inflammation, changing the macrophage phenotype (52, 53). The data reported in our work highlighted that PBDE-47 was able to modulate the upload of a large set of miRNAs in the sEVs^PBDE+LPS^, some of them known to be involved in regulation of macrophage polarization (54–56). Our approach allowed us to define a miRNA EV-associated signature upon stimulation with PBDE-47 in THP-1 derived macrophage-like cells.

To understand the way bystander cells may sense the sEVs^PBDE+LPS^, we decided to further investigate the functional role of these sEVs by setting up a culture assay in which PMA-differentiated naïve M(0) resting THP-1 cells were incubated with the purified sEVs, looking at their effect on 1) the intracellular expression levels of selected miRNAs, 2) the gene expression of cytokines markers of M1 or M2 polarization, and 3) the expression of markers of macrophage polarization by flow cytometry.

Microarray analysis showed that sEVs^PBDE+LPS^ can modulate intracellular PMA-differentiated M(0) THP-1 miRNA expression, inducing the downregulation of miR-194-5p, miR-374a-5p, miR-23a-3p, miR-126-3p, miR-195-5p, miR-30d-5p, miR-222-3p, miR-186-5p, miR-196b-5p, miR-140-3p, and miR-100-5p, and the upregulation of -miR-142-3p. These set of data were analyzed *in silico* to try to identify molecular pathways regulated by the set of twelve PBDE-47-modulated miRNAs. Pathway enrichment analysis followed by clustering of the most representative ones identified 16 pathways containing genes with reference to the immune response and macrophage polarization (**Table 5**). The Graphical Summary reported within **Figure 4** provides a quick overview of the major biological identified pathways and how they relate to each other. In particular, Nuclear Receptors (NRs) are key regulators of innate immune responses and tissue homeostasis, influencing immune tolerance and the resolution of inflammation *via* the modulation of macrophage function (57). NRs are key drivers of macrophage polarization, attenuating inflammatory responses and promoting alternative activation (58). The PI3K-AKT pathway is an intracellular signal transduction pathway that promotes metabolism, proliferation, cell survival, growth, and angiogenesis in response to extracellular signals. It is widely accepted that PI3K/AKT signaling regulates the effector responses of macrophages (59), displaying a direct effect on macrophage polarization (60). Moreover, the PI3 Kinase/AKT pathway is also a complex network of adaptors and transducers connected to other pathways highlighted in our *in silico* analysis, such as the c-Met involved in cytoskeleton dynamics, cell scattering and migration, cell adhesion and invasion (61). The VEGF/VEGFR axis has different biological effects on macrophage infiltration/activation (62). A very well characterized pathway downstream from VEGFR2 is the above-described PIP3-AKT signaling, which is also regulated by the FLT-3 (or FMS-like tyrosine kinase 3) pathway that drives the development and proliferation of hematopoietic stem cell (HSC) and B cell progenitors (63, 64). Among the different downstream targets of AKT, it is also possible to find BAD (Bcl2-associated agonist of cell death) and endothelial nitric oxide synthase (eNOS), which have also been identified in our pathway analysis (65). Interleukin (IL)-4 and IL-13 are structurally and functionally related cytokines not only involved in immune function but also in pregnancy, fetal development, mammary development, and lactation (66). Both cytokines are more widely known for their role in the pathogenesis of atopy, asthma, pulmonary fibrosis, and cancer, but they also can induce “alternative activation” of macrophages, inducing an anti-inflammatory phenotype. This signaling plays a key role in the T helper 2 response, mediating anti-parasitic effects and influencing wound healing (67). RUNX1 is a transcription factor master regulator of hematopoiesis which is able to determine the fate of the myeloid lineage during normal stem cell development. The RUNX1 transcription factor controls the growth and survival of macrophages, and it is generally accepted that RUNX1 reshapes the epigenetic landscape at the onset of hematopoiesis (68, 69). Finally, the preferential expression of CD28 mRNA in monocyte-derived macrophages generated in the presence of M-CSF (monocyte-derived macrophages [M-MØ]), or in the presence of GM-CSF (pro-inflammatory GM-MØ), demonstrated that its expression by human macrophages is mediated by cytokines and factors that determine the acquisition of pro- or anti-inflammatory profiles (70, 71).

Starting from this scenario, we further sought to analyze the regulatory role of macrophage sEVs^PBDE+LPS^ on resting PMA-differentiated (M0) THP-1 macrophage-like cells, looking at both the expression of IL-6, TNF-α and TGF-β gene expression and at the differential expression of known surface markers of macrophage polarization by means of flow cytometry. Real Time PCR analysis showed that treatment with sEVs^PBDE+LPS^ did not induce the expression of cytokines markers of M1 or M2 polarization. Then, we evaluated surface antigens such as HLA-DR, that regulates macrophage phenotypic transformation; CD80, that provides costimulatory signals required for development of antigen-specific immune responses; and CCR7 that is not only a marker for M1 macrophage polarization (72) but also correlates with enhanced phagocytosis of antigens. HLA-DR expression was decreased upon *in vitro* culture with macrophage sEVs^PBDE+LPS^, demonstrating that PBDE-47 treatment induced a reduction in the expression of such marker when compared to the control sEVs^DMSO+LPS^. On the other hand, using the same experimental conditions, no PBDE-47 specific modulation was observed for the CD80 and CCR7 antigens. Indeed, data observed on HLA-DR were strengthened by MFI analysis, whereas non-significant results were confirmed for CD80 and CCR7 expression.

M(0) polarization has been widely studied with the intention of finding more precise polarization markers. Recently, CD38 and CD209 were identified as markers of polarized macrophages. In particular, CD38 is strongly up-regulated in murine M1 macrophages, while its expression is down-regulated in M2 macrophages compared with that in M0 macrophages, which suggests that CD38 is the exclusive expression pattern of M1 macrophages (73). CD209 on macrophages recognizes and binds to high-mannose type N-glycans, a class of PAMPs (pathogen associated molecular patterns) commonly found on viruses, bacteria, and fungi activating phagocytosis (74). In our experimental set-up, we observed that culturing sEVs^PBDE+LPS^ on M(0) THP-1 macrophage-like cells induced changes in CD209 expression, with a statistically significant reduction in the sEVs^PBDE+LPS^ treated cells versus control sEVs^DMSO+LPS^ samples; meanwhile no changes in CD38 expression were observed. Moreover, the observed CD209 decrease in LPS-stimulated cells was in accordance with already published data (41).

The extraordinary adaptability of macrophages in response to environmental signals is larger than the original M1/M2 dichotomy, as the studies on TAMs and genome-wide studies have already shown. Transcriptional and epigenetic modifications identified that differences in noncoding-RNAs, histone modifications, and DNA methylation can strongly affect the fate of macrophages as well as the type of the inflammatory response (75–77). Plasticity is a peculiar trait of macrophages therefore an excessive or inadequate activation may lead to dysregulations during inflammatory and autoimmune responses (78). In our report, we describe that PBDE-47 can modulate a large set of the miRNA cargo loaded on sEVs from PBDE-47 treated M(LPS) THP-1 macrophage-like cells and downstream, by means of these vesicles, impact the expression of several intracellular miRNAs in naïve resting M(0) THP-1 macrophage-like cells. Furthermore, bioinformatic tools suggest that sEVs’ treatment can affect relevant pathways involved in the activity and polarization of macrophages. This analysis was confirmed by Real Time PCR and by immunostaining data where naïve resting M(0) THP-1 derived macrophages cultured with sEVs^PBDE+LPS^ did not display the transcriptional activation of both M1 and M2 cytokine markers (IL-6, TNF-α and TGF-β) and may have impaired the ability to make immunological synapses and present antigens downregulating the expression of HLA-DR and CD209 antigens.

This study displays some limitations since experiments were performed with the PMA differentiated THP-1 cell line which, upon *in vitro* differentiation, acquire a macrophage-like phenotype which mimics primary human macrophages. Ex vivo human monocyte-derived macrophages (MDMs) are the most commonly used precursors for generating macrophages in *in vitro* differentiation assays, however the use of MDMs present several technical issues regarding donor-dependent variability and ability to proliferate to a significant extent. Conversely, the THP-1 cell line is a commonly used source for human macrophages that can be easily differentiated and expanded *in vitro* whose single genetic background should minimize the variability of cell phenotype allowing a better standardization of the conditions for the assays and allowing cell culture conditions for large purification of sEVs (79, 80). Furthermore, the use of sEVs does not indicate which component(s) of the sEVs^PBDE+LPS^ cargo play(s) a major role in mediating epigenetic changes and immunomodulate macrophage activity although several reports established that miRNAs cargo are key molecular switches in macrophage activation therefore, we specifically focused our attention on these molecules (81). Moreover, it is relevant to mention that our assays were performed using a concentration of the environmentally pollutant and of the sEVs similar to real life conditions making our study reliable for the analysis of the interactions between environmental pollution and innate immune response (81).

In conclusion, this and our previous studies demonstrated that PBDE-47 can have diverse action mechanisms with a direct immunotoxic effects on macrophage, impairing the secretion of proinflammatory cytokines in M(LPS) macrophages (17), affecting sEVs biogenesis (16) and their miRNA cargo. Furthermore, we demonstrate that sEVs’ cargo is regulated with purposeful consequences toward downstream events and target cells. In fact, purified sEVs^PBDE+LPS^ are capable to modulate intracellular miRNAs involved in several pathways relevant for macrophages differentiation and affect the expression of surface markers in naïve resting M(0) macrophages confirming the immunotoxicity of this compound. Finally, these observations further suggest for the integration of extracellular vesicles into exposure science and toxicology for their capability of influencing nearby and distal cells upon pollutant treatments.

## Data availability statement

The raw data supporting the conclusions of this article will be made available by the authors, without undue reservation.

## Author contributions

PC, FC, SM, AB, AU reviewed and analyzed the data. VL, AL, NA, AF, ELP, performed all the experiments. All authors contributed to the article and approved the submitted version.
