# Supplementary figures and images for: Impact of the flame retardant 2,2’4,4’-tetrabromodiphenyl ether (PBDE-47) in THP-1 macrophage-like cell function via small extracellular vesicles
Source: Front Immunol. 2023 Jan 6;13:1069207. doi: 10.3389/fimmu.2022.1069207 (PMC9852912; doi:10.3389/fimmu.2022.1069207)

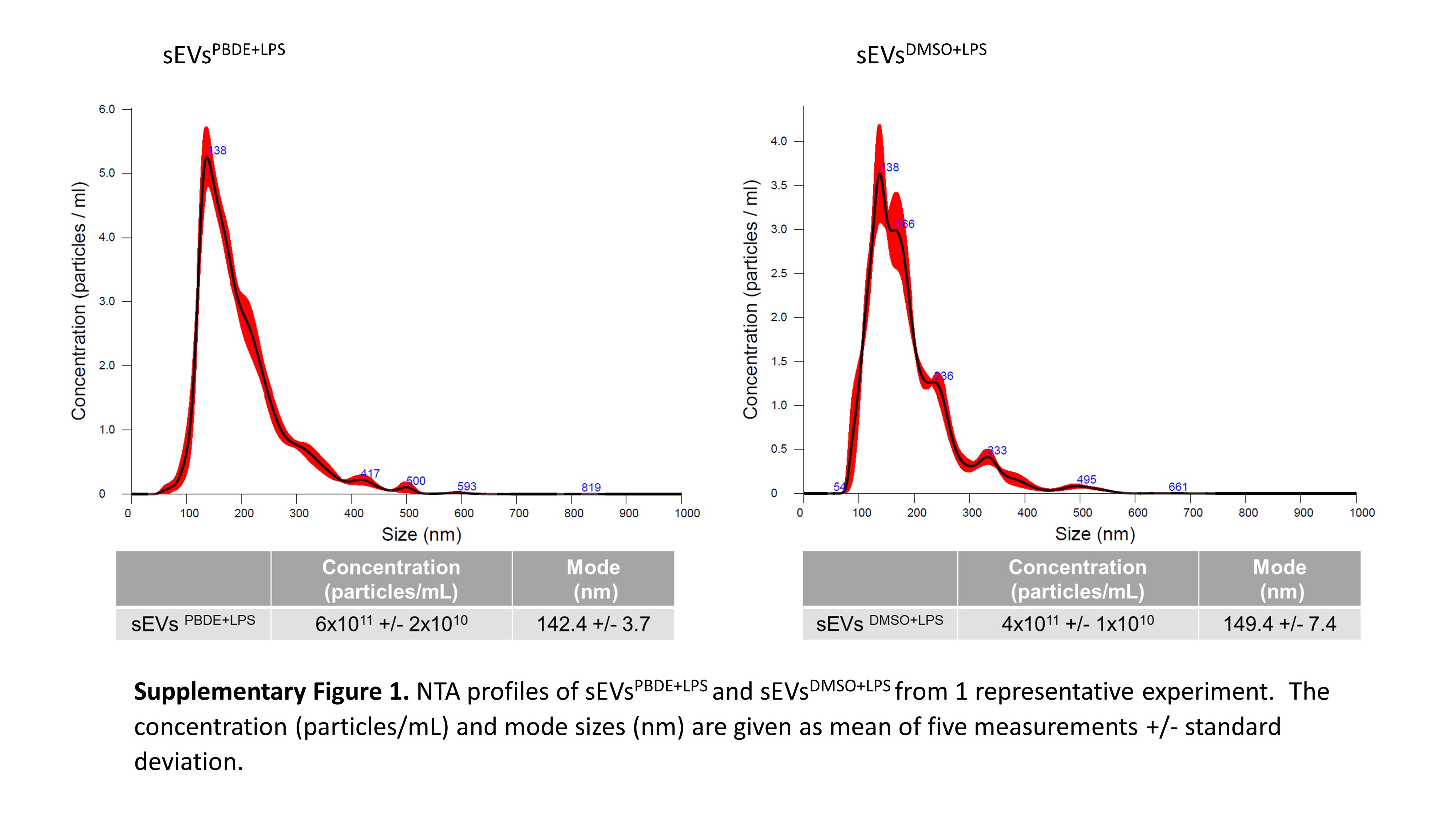

Supplement: Supplementary file 1 [file Image_1.jpeg]

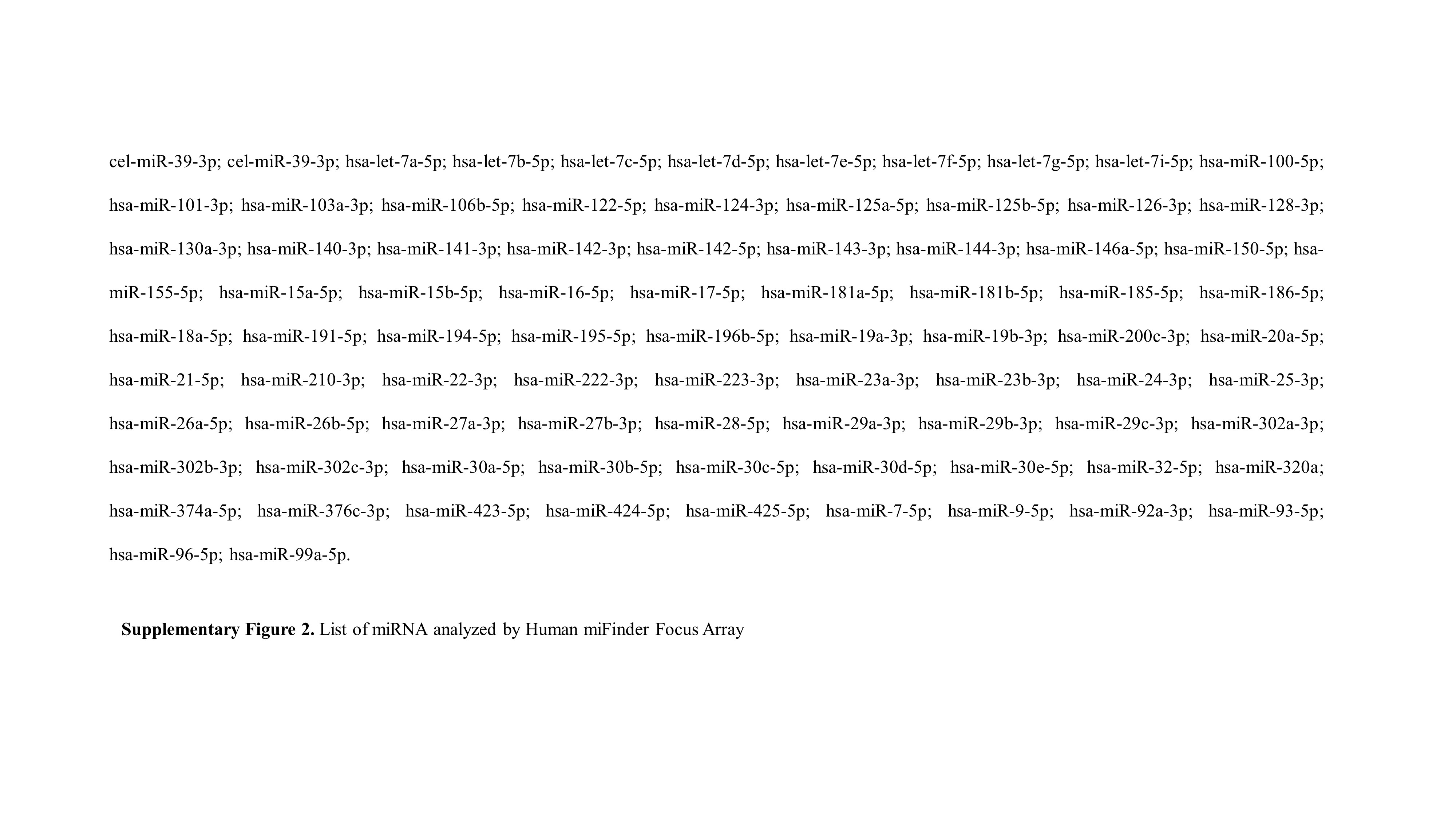

Supplement: Supplementary file 2 [file Image_2.jpeg]

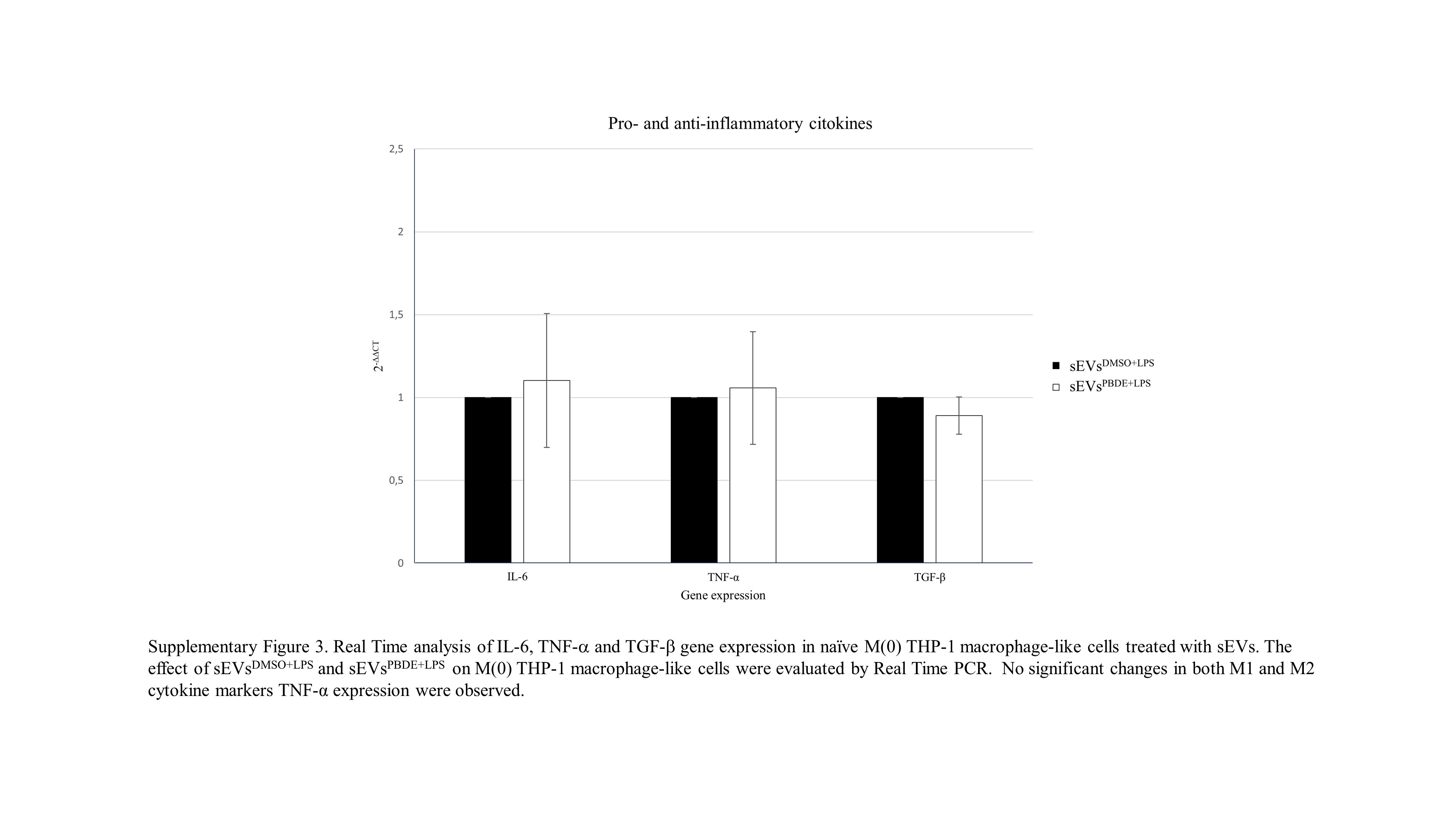

Supplement: Supplementary file 3 [file Image_3.jpg]
